# *Similiboletinus tomentopileatus* gen. et sp. nov. from Tropical China, a New Boletoid Taxon Based on Morphological and Molecular Data

**DOI:** 10.3390/jof12020145

**Published:** 2026-02-16

**Authors:** Sipeng Jian, Xinjing Xu, Feng Gao, Yiwei Fang, Tianwei Yang, Jing Liu, Wenzhu Ai, Chunxia Zhang, Yanchun Li

**Affiliations:** 1 Yunnan Institute of Tropical Crops, Jinghong 666100, China; a1464993369@gmail.com (S.J.); 15198689253@163.com (X.X.); gaofeng19860704@outlook.com (F.G.); fangyiwei11@163.com (Y.F.); yangtianweizj@126.com (T.Y.); ljxsbn@126.com (J.L.); zhuge68168@163.com (W.A.); 2 CAS Key Laboratory for Plant Diversity and Biogeography of East Asia, Kunming Institute of Botany, Chinese Academy of Sciences, Kunming 650201, China; 3 Yunnan Key Laboratory for Fungal Diversity and Green Development, Kunming 650201, China

**Keywords:** Boletaceae, Suillelloideae, multigene phylogeny, new taxa, taxonomy

## Abstract

Boletaceae, a family of profound ecological influence and economic value, has been the subject of prolific taxonomic research over the past decade, with new taxa continually being discovered. In this study, *Similiboletinus tomentopileatus* gen. et sp. nov., collected from a tropical region of China, is proposed based on integrative morphological and phylogenetic analyses. Phylogenetic analyses of a multigene dataset (LSU-*rpb2*-*tef1-α*-*atp6*) strongly support the position of *Similiboletinus* within the Suillelloideae. Morphologically, it is characterized by a tomentose and yellow–brown pileus, a subdecurrent hymenophore, unchanging context when injured, a boletoid hymenophoral trama, a cutis-type pileipellis, and broadly ellipsoid to ovoid basidiospores. The identification of *Similiboletinus* further underscores the striking and largely unexplored diversity of Boletaceae within tropical China.

## 1. Introduction

The family Boletaceae Chevall. (Boletales, Agaricomycetes) plays vital roles in diverse ecosystems, primarily owing to its ectomycorrhizal symbiotic abilities [1,2]. Additionally, it holds significant economic values in the market, largely due to the edible value of its members [3,4]. Morphologically, most fungi in Boletaceae are stipitate-pileate (i.e., boletoid or phylloporoid) and rarely gasteroid [5,6,7]. They are typically characterized by fleshy context and tubulose, lamellate or loculate hymenophore [8,9,10]. Recent phylogenetic studies have classified Boletaceae into eight subfamilies: Austroboletoideae G. Wu & Zhu L. Yang, Boletoideae Singer, Chalciporoideae G. Wu & Zhu L. Yang, Leccinoideae G. Wu & Zhu L. Yang, Phylloboletelloideae Dentinger et al., Suillelloideae Dentinger et al., Xerocomoideae Singer, and Zangioideae G. Wu et al. [8,11]. To date, approximately 110 genera and 1200 species have been reported in this family [11,12].

Among the various subfamilies of Boletaceae, the Suillelloideae has attracted particular attention due to its remarkable morphological diversity, including gasteroid, secotioid and stipitate-pileate forms. It was recently erected by Tremble et al. [13] and its phylogenetic position was further consolidated using whole-genome sequencing data [11]. Thus, the formal name Suillelloideae has replaced the provisional designation “*Pulveroboletus* Group”, which was proposed by Wu et al. [8] to accommodate several distinct genera. Currently, 27 genera are recognized under this subfamily, half of which (13 genera) have been erected by Chinese researchers, namely *Acyanoboletus*, *Amoenoboletus*, *Baorangia*, *Crocinoboletus*, *Erythrophylloporus*, *Hemilanmao*, *Hongoboletu*, *Lanmaoa*, *Pseudobaorangia*, *Rubroboletus*, *Rubroleccinum*, *Rufoboletus*, and *Rugiboletus* (Table 1).

In China, particularly in Yunnan Province, boletes are highly valued for their exceptional organoleptic qualities and medicinal properties, contributing substantially to local economies [12,15,32]. Moreover, the region’s microhabitats, shaped by variations in altitude and precipitation, along with the abundance of host plants (primarily Fagaceae and Pinaceae), have facilitated species diversification within Boletaceae [36,37,38]. Although numerous genera and species of Boletaceae have been documented in this region [4,12,15,39], this biodiversity hotspot continues to harbor novel genera and taxa awaiting discovery. Recent species surveys conducted in tropical areas of Yunnan have yielded several noteworthy specimens. Subsequent morphological and molecular phylogenetic analyses indicate that these specimens represent a novel genus and species, which could contribute significantly to understanding the diversity and evolution of Boletaceae in this biodiversity hotspot. A detailed description of this new taxon is provided herein.

## 2. Materials and Methods

### 2.1. Sampling and Morphological Studies

The collection information of voucher specimens and the sequences used for phylogenetic analyses are provided in Table 2. Color codes (hex triplets) from ColorHexa (https://www.colorhexa.com) were used to represent the color of basidiomata. Each code consists of characters ranging from “a” to “f” and “0” to “9”, with each pair corresponding to the red, green, and blue components of the color. The morphological classification framework for Boletaceae followed the criteria established by Vellinga et al. [40], Wu et al. [9], and Li and Yang [7]. All voucher specimens have been deposited in the Cryptogamic Herbarium (HKAS) of the Herbaria of Kunming Institute of Botany, Chinese Academy of Sciences (KUN).

Sections of dried basidiomata were rehydrated in 5-10% KOH solution and occasionally stained with 1% Congo red to improve visibility. The Melzer’s reagent was also used to test the amyloidity or dextrinoidity of the basidiospores. The notation “[*n*/*m*/*p*]” indicates that *n* basidiospores were measured from *m* basidiomata of *p* specimens. Measurements of basidiospores are presented as (a–)b–c(–d); the range b–c stands for a minimum of 90% of the measured values, while a and d (given in parentheses) represent the extreme values. The mean length and width of basidiospore (±standard deviation) are abbreviated as L_m_ and W_m_, respectively. The symbol Q refers to the “length/width ratio” of a basidiospore in lateral view, with Q_m_ representing the average Q of all basidiospores (± sample standard deviation). For scanning electron microscopy (SEM), tiny hymenophore fragments from dried specimens were mounted on aluminum stubs using double-sided adhesive tape, coated with gold-palladium, and then observed under a Hitachi S4800 (Hitachi high-tech corporation, Ibaraki, Japan) scanning electron microscope to examine the basidiospore ornamentation.

### 2.2. DNA Extraction, PCR Amplification, and DNA Sequencing

In this study, four nuclear loci were employed, comprising one non-protein-coding and three protein-coding genes. They are the large subunit ribosomal RNA gene (LSU), the RNA polymerase II second largest subunit (*rpb2*), the translation elongation factor 1-alpha gene (*tef1-α*), and the mitochondrial ATPase subunit 6 (*atp6*). Detailed primer information (LR0R/LR5, bRPB2-6F/bRPB2-7.1R, EF1-983F/EF1-1953R, and ATP6-3/ATP6-6R) followed the references cited in Wu et al. [8], Li et al. [41], and Jian et al. [42]. Furthermore, the internal transcribed spacer (ITS) region (ITS1-5.8S-ITS2) was sequenced with the primers ITS5/ITS4 for subsequent BLAST queries (https://blast.ncbi.nlm.nih.gov/) and phylogenetic placement.

DNA was extracted from collected samples and herbarium specimens using the cetyltrimethylammonium bromide (CTAB) method described by Doyle and Doyle [43]. Polymerase chain reaction (PCR) amplification was carried out following the procedure implemented by Wu et al. [8] and Li and Yang [7]. The PCR products were purified via gel extraction and subsequently sequenced on an ABI-3730-XL DNA analyzer (Applied Biosystems, Foster City, CA, USA) using the same primers as employed in amplification. When PCR products could not be sequenced directly, they were cloned into a PMD18-T vector (Takara, Shiga, Japan), then amplified, and sequenced using the standard primers M13F and M13R. All newly generated sequences are indicated in bold in Table 2 and deposited in the GenBank database.

**Table 2:** Voucher specimens and GenBank accession numbers for sequences used in phylogenetic analyses. E or H in parentheses refers to the epitype or holotype specimen. Sequences newly generated in this study are shown in bold.

Species	Collection No.	Country	GenBank Accession Numbers				References
			LSU	*rpb2*	*tef1-α*	*atp6*	
*Acyanoboletus controversus*	HKAS101248	China	OQ888715	OQ873491	OQ873452	-	[12]
*Acyanoboletus controversus* (H)	HKAS126560	China	OQ888714	OQ873490	OQ873451	-	[12]
*Acyanoboletus dissimilis* (H)	ZT14030	Malaysia	OQ888716	OQ873492	OQ873453	-	[12]
*Amoenoboletus granulopunctatus*	HKAS56280	China	KF112418	KF112708	KF112265	-	[8]
*Amoenoboletus granulopunctatus*	MHHNU9490	China	MW520186	MW560081	MW566747	-	[14]
*Amoenoboletus granulopunctatus*	HKAS86007	China	MW520187	MW560079	MZ741478	-	[14]
*Amoenoboletus granulopunctatus*	HKAS80250	China	MW520185	MW560080	MW566746	-	[14]
*Amoenoboletus mcrobbii*	PDD 97418	New Zealand	MW520184	-	MW566744	-	[14]
*Amoenoboletus miraculosus* (H)	Z-ZT 14046	Malaysia	MW520188	-	MW566745	-	[14]
*Baorangia major*	OR0486	China	-	MG897443	MG897433	MG897423	[44]
*Baorangia pseudocalopus*	HKAS75081	China	KF112356	KF112678	KF112168	-	[8]
*Baorangia rufomaculata*	BOTH4144	USA	-	MG897435	MG897425	MG897415	[44]
*Boletus bainiugan*	HKAS52235	China	KF112457	KF112705	KF112203	-	[8]
*Boletus* sp.	JD0693	Burundi	-	MH645599	MH645591	MH645583	[17]
*Buchwaldoboletus lignicola*	VDKO1140	Belgium	-	MH614756	MH614710	MH614661	[17]
*Buchwaldoboletus xylophilus*	FHMU5930	China	MW783417	MW820939	MW897330	-	[45]
*Buchwaldoboletus xylophilus*	FHMU5933	China	MW783423	MW820945	MW897336	-	[45]
*Butyriboletus appendiculatus*	VDKO0193b	Belgium	-	MG212624	MG212582	MG212537	[46]
*Butyriboletus* cf. *roseoflavus*	OR0230	China	-	KT824007	KT824040	KT823974	[47]
*Butyriboletus pseudoregius*	VDKO0925	Belgium	-	MG212625	MG212583	MG212538	[46]
*Butyriboletus pseudospeciosus* (H)	HKAS63513	China	KT990541	KT990380	KT990743	-	[9]
*Butyriboletus roseoflavus*	HKAS54099	China	KY418892	KF739703	KF739779	-	[8]
*Butyriboletus roseopurpureus*	BOTH4497	USA	-	MG897438	MG897428	MG897418	[44]
*Butyriboletus subsplendidus*	HKAS50444	China	KT990540	KT990379	KT990742	-	[9]
*Butyriboletus yicibus*	HKAS55413	China	KF112338	KF112674	KF112157	-	[8]
*Cacaoporus pallidicarneus* (H)	SV0221	Thailand	-	MK372286	MK372273	MK372262	[17]
*Cacaoporus tenebrosus*	SV0224	Thailand	-	MK372291	MK372278	MK372267	[17]
*Cacaoporus tenebrosus* (H)	SV0223	Thailand	-	MK372290	MK372277	MK372266	[17]
*Caloboletus* aff. *calopus*	HKAS74739	China	KF112335	KF112667	KF112166	-	[8]
*Caloboletus calopus*	ADK4087	Belgium	KJ184554	KP055030	KJ184566	MG212539	[48]
*Caloboletus inedulis*	BOTH3963	USA	-	MG897434	MG897424	MG897414	[44]
*Caloboletus panniformis*	HKAS55444	China	KF112334	KF112666	KF112165	-	[8]
*Caloboletus radicans*	VDKO1187	Belgium	-	MG212626	MG212584	MG212540	[46]
*Caloboletus* sp.	HKAS53353	China	KF112410	KF112668	KF112188	-	[8]
*Caloboletus* sp.	OR0068	Thailand	-	MH614757	MH614711	MH614662	[17]
*Caloboletus yunnanensis* (H)	HKAS69214	China	KJ184556	KT990396	KJ184568	-	[48]
*Chalciporus africanus*	JD0517	Cameroon	-	KT823996	KT824029	KT823963	[47]
*Chalciporus piperatus*	VDKO1063	Belgium	-	MH614759	MH614713	MH614664	[17]
*Costatisporus cyanescens* (H)	Henkel9061	Guyana	LC053662	LC053664	-	-	[19]
*Crocinoboletus* cf. *laetissimus*	OR0576	Thailand	-	KT824008	KT824041	KT823975	[47]
*Crocinoboletus laetissimus*	FHMU2030	China	MK850935	MK850944	MK850948	-	[12]
*Crocinoboletus rufoaureus*	HKAS 53424	China	KF112435	KF112710	KF112206	-	[12]
*Cupreoboletus poikilochromus*	GS10070	Italy	KT157060	KT157068	KT157072	-	[21]
*Cupreoboletus poikilochromus*	GS11008	Italy	KT157059	KT157067	KT157071	-	[21]
*Cyanoboletus bessettei*	ARB1393A	USA	MW662571	MW737457	MW737482	-	[49]
*Cyanoboletus brunneoruber* (H)	HKAS80579_1	China	KT990568	KT990401	KT990763	-	[9]
*Cyanoboletus cyaneitinctus*	Farid920	USA	MW662579	MW737465	-	-	[49]
*Cyanoboletus fagaceophilus* (H)	HKAS126556	China	OQ888718	OQ873494	OQ873455	-	[12]
*Cyanoboletus instabilis*	HKAS59554	China	KF112412	KF112698	KF112186	-	[8]
*Cyanoboletus pulverulentus*	MG628a	Italy	KT157064	KT157069	KT157073	-	[21]
*Cyanoboletus sinopulverulentus*	HKAS59609	China	KF112366	KF112700	KF112193	-	[8]
*Cyanoboletus* sp.	HKAS59418	China	KT990570	KT990403	KT990765	-	[9]
*Cyanoboletus* sp.	HKAS76850	China	KF112343	KF112697	KF112187	-	[8]
*Cyanoboletus* sp.	HKAS90208_1	China	KT990571	KT990404	KT990766	-	[9]
*Cyanoboletus* sp.	OR0322	Thailand	-	MH614768	MH614722	MH614673	[17]
*Erythrophylloporus aurantiacus*	REH7271	Costa Rica	-	MH614761	MH614715	MH614666	[50]
*Erythrophylloporus cinnabarinus*	GDGM46541	China	MH374043	MH374034	MH378803	-	[23]
*Erythrophylloporus cinnabarinus* (H)	GDGM70536	China	MH374045	MH374035	MH378802	-	[23]
*Erythrophylloporus fagicola*	Garay215	Mexico	-	MH614762	MH614716	MH614667	[50]
*Erythrophylloporus paucicarpus*	OR0689	Thailand	-	MH614763	MH614717	MH614668	[50]
*Erythrophylloporus paucicarpus*	OR1135	Thailand	-	MH614764	MH614718	MH614669	[50]
*Erythrophylloporus paucicarpus* (H)	OR1151	Thailand	-	MH614765	MH614719	MH614670	[50]
*Erythrophylloporus suthepensis*	OR0615B	Thailand	-	MH614766	MH614720	MH614671	[50]
*Erythrophylloporus suthepensis* (H)	SV0236	Thailand	-	MH614767	MH614721	MH614672	[50]
*Exsudoporus floridanus* (E)	FLAS-F-59069	USA	OL960488	OL960503	OL960496	-	[51]
*Exsudoporus* *frostii*	NY815462	USA	JQ924342	KF112675	KF112164	-	[8]
*Exsudoporus* *permagnificus*	MG829	Italy	-	OL960510	OL960502	-	[51]
*Exsudoporus* *ruber*	HKAS106891	China	MN930518	MT063120	MT063123	-	[52]
*Exsudoporus* *ruber*	HKAS103513	China	MN930519	MT063121	MT063124	-	[52]
*Gymnogaster boletoides*	NY01194009	Australia	KT990572	KT990406	KT990768	-	[9]
*Gymnogaster boletoides*	REH9455	Australia	JX889673	-	JX889683	-	[35]
*Hemilanmaoa reticulatistipitata*	HMJAU60053	China	OP380696	OP495815	OP495817	-	[26]
*Hemilanmaoa reticulatistipitata* (H)	HMJAU60052	China	OP380695	OP495814	OP495816	-	[26]
*Hongoboletus* sp.	OR1002	Thailand	-	MH645601	MH645593	MH645585	[17]
*Hongoboletus ventricosus*	TNS-F-44611	Japan	OQ888732	OQ873507	-	-	[12]
*Hongoboletus ventricosus*	TNS-F-44612	Japan	OQ888733	OQ873508	-	-	[12]
*Hongoboletus ventricosus*	HKAS122793	China	OM219809	OM562220	OM562214	-	[12]
*Hongoboletus ventricosus*	HKAS63598	China	KF112317	KF112663	KF112152	-	[12]
*Imperator torosus*	MB000258	Germany	-	MW560082	MW566748	-	[14]
*Lanmaoa angustispora*	HKAS74759	China	KM605140	KM605178	KM605155	-	[15]
*Lanmaoa asiatica*	HKAS63516	China	KT990584	KT990419	KT990780	-	[9]
*Leccinellum crocipodium*	VDKO1006	Belgium	-	KT824021	KT824054	KT823988	[47]
*Leccinum variicolor*	VDKO0844	Belgium	-	MG212636	MG212594	MG212550	[46]
*Neoboletus brunneissimus*	HKAS50538	China	KM605138	KM605173	KM605150	-	[15]
*Neoboletus brunneissimus*	HKAS52660	China	KF112314	KF112650	KF112143	-	[8]
*Neoboletus brunneissimus*	HKAS57451	China	KM605137	KM605172	KM605149	-	[15]
*Neoboletus brunneissimus*	OR0249	China	-	MG212637	MG212595	MG212551	[46]
*Neoboletus brunneorubrocarpus*	HKAS126559	China	OQ888720	OQ873496	OQ873457	-	[12]
*Neoboletus brunneorubrocarpus* (H)	HKAS76660	China	KF112328	KF112731	KF112180	-	[8]
*Neoboletus erythropus*	VDKO0690	Belgium	-	KT824015	KT824048	KT823982	[47]
*Neoboletus ferrugineus*	HKAS77617	China	KT990595	KT990430	KT990788	-	[9]
*Neoboletus ferrugineus* (H)	HKAS77718	China	KT990596	KT990431	KT990789	-	[9]
*Neoboletus flavidus*	HKAS58724	China	KF739686	KU974145	KU974137	-	[9]
*Neoboletus flavidus* (H)	HKAS59443	China	KF739684	KU974144	KU974136	-	[9]
*Neoboletus hainanensis*	HKAS63515	China	KT990614	KT990449	KT990808	-	[9]
*Neoboletus hainanensis*	HKAS74880	China	KT990597	KT990432	KT990790	-	[9]
*Neoboletus hainanensis*	HKAS90209	China	KT990615	KT990450	KT990809	-	[9]
*Neoboletus hainanensis*	HKAS59469	China	KF112359	KF112669	KF112175	-	[8]
*Neoboletus junquilleus*	AF2922	France	-	MG212638	MG212596	MG212552	[46]
*Neoboletus magnificus*	HKAS54096	China	KF112324	KF112654	KF112149	-	[8]
*Neoboletus magnificus*	HKAS74939	China	KF112320	KF112653	KF112148	-	[8]
*Neoboletus multipunctatus*	HKAS76851	China	KF112321	KF112651	KF112144	-	[8]
*Neoboletus multipunctatus*	OR0128	Thailand	-	MH614781	MH614734	MH614686	[17]
*Neoboletus obscureumbrinus*	OR0553	Thailand	-	MK372294	MK372282	MK372271	[17]
*Neoboletus obscureumbrinus*	HKAS63498	China	KT990598	KT990433	KT990791	-	[9]
*Neoboletus obscureumbrinus*	HKAS77774	China	-	KT990434	KT990792	-	[9]
*Neoboletus obscureumbrinus*	HKAS89014	China	KT990599	KT990435	KT990793	-	[9]
*Neoboletus obscureumbrinus*	HKAS89027	China	KT990600	KT990436	KT990794	-	[9]
*Neoboletus rubriporus*	HKAS57512	China	KF112327	KF112656	KF112151	-	[8]
*Neoboletus rubriporus* (H)	HKAS83026	China	KT990601	KT990437	KT990795	-	[9]
*Neoboletus sanguineoides*	HKAS57766	China	KT990605	KT990440	KT990799	-	[9]
*Neoboletus sanguineoides*	HKAS55440	China	KF112315	KF112652	KF112145	-	[8]
*Neoboletus sanguineoides* (H)	HKAS74733	China	KT990606	KT990441	KT990800	-	[9]
*Neoboletus sanguineus* (H)	HKAS80823	China	KT990608	KT990442	KT990802	-	[9]
*Neoboletus tomentulosus*	HKAS77656	China	KT990611	KT990446	KT990806	-	[9]
*Neoboletus tomentulosus*	HKAS53369	China	KF112323	KF112659	KF112154	-	[8]
*Neoboletus venenatus*	HKAS57489	China	KF112325	KF112665	KF112158	-	[8]
*Neoboletus venenatus*	HKAS63535	China	KT990613	KT990448	KT990807	-	[9]
*Pulveroboletus brunneopunctatus*	HKAS74926	China	KT990621	KT990456	KT990815	-	[9]
*Pulveroboletus fragrans* (H)	OR0673	Thailand	-	KT824010	KT824043	KT823977	[47]
*Pulveroboletus macrosporus*	HKAS58860	China	KF112408	KF112714	KF112263	-	[8]
*Pulveroboletus ravenelii*	REH2565	USA	-	KU665637	KU665636	KU665635	[47]
*Pulveroboletus subrufus*	N.K.Zeng1857	China	KX453837	KX453841	KX453855	-	[53]
*Rubroboletus esculentus*	HKAS68679	China	KF112333	KF112662	KF112147	-	[8]
*Rubroboletus flavus* (H)	HKAS90906	China	OQ888722	OQ873497	OQ873459	-	[12]
*Rubroboletus latisporus*	HKAS63517	China	KP055022	KP055028	KP055019	-	[31]
*Rubroboletus latisporus* (H)	HKAS80358	China	NG_059540	KP055029	KP055020	-	[31]
*Rubroboletus legaliae*	VDKO0936	Belgium	-	KT824018	KT824051	KT823985	[47]
*Rubroboletus rhodosanguineus*	BOTH4263	USA	-	MG897436	MG897426	MG897416	[44]
*Rubroboletus rhodoxanthus*	HKAS84879	Germany	KT990637	KT990468	KT990831	-	[9]
*Rubroboletus satanas*	VDKO0968	Belgium	-	KT824019	KT824052	KT823986	[47]
*Rubroboletus serpentiformis*	HKAS126557	China	OQ888723	OQ873498	OQ873460	-	[12]
*Rubroboletus sinicus*	HKAS68620	China	KF112319	KF112661	KF112146	-	[8]
*Rubroboletus sinicus*	HKAS56304	China	KJ605673	KP055031	KJ619483	-	[31]
*Rubroleccinum latisporus*	FHMU7698	China	PQ325253	PQ330108	PQ330106	-	[32]
*Rubroleccinum latisporus* (H)	FHMU7699	China	PQ325254	PQ330109	PQ330107	-	[32]
*Rufoboletus hainanensis*	N.K.Zeng1197	China	KU961651	KU961658	-	-	[54]
*Rufoboletus hainanensis*	N.K.Zeng2418	China	KU961652	KX453856	KU961656	-	[54]
*Rugiboletus brunneiporus*	HAKS 83210	China	KM605132	KM605171	KM605145	-	[15]
*Rugiboletus brunneiporus* (H)	HKAS 83209	China	KM605134	KM605168	KM605144	-	[15]
*Rugiboletus caeruloruber*	HMJAU68163	China	-	OR566424	-	-	[55]
*Rugiboletus caeruloruber*	HMJAU68164	China	-	-	OR566419	-	[55]
*Rugiboletus caeruloruber* (H)	HMJAU68165	China	OR554014	OR566425	OR566420	-	[55]
*Rugiboletus extremiorientalis*	OR0406	Thailand	-	MG212647	MG212607	MG212562	[46]
*Rugiboletus extremiorientalis*	HKAS 76663	China	KM605135	KM605170	KM605147	-	[15]
***Similiboletinus tomentopileatus***	**HKAS152700**	**China**	**PX843739**	**PX854051**	**PX880578**	**-**	**This study**
***Similiboletinus tomentopileatus***	**HKAS152701**	**China**	**PX843740**	**PX854052**	**PX880579**	**PX880583**	**This study**
***Similiboletinus tomentopileatus*** **(H)**	**HKAS152702**	**China**	**PX843741**	**PX854053**	**PX880580**	**PX880584**	**This study**
***Similiboletinus tomentopileatus***	**HKAS152703**	**China**	**PX843742**	**PX854054**	**PX880581**	**PX880585**	**This study**
*Singeroboletus himalayanus*	KD 22-018	India	PP133252	PP157641	PP188020	-	[56]
*Singeroboletus himalayanus* (H)	KD 23-006	India	PP133251	PP157640	PP188019	-	[56]
*Singerocomus guadelupae*	ACM 527	Brazil	KY926776	-	-	-	[57]
*Singerocomus guadelupae*	ACM 1275	Brazil	KY926777	-	OM160563	-	[57]
*Singerocomus inundabilis*	TWH9199	Guyana	LC043087	LC043089	MH645596	MH645588	[34]
*Singerocomus inundabilis*	TWH8408	Guyana	HQ161863	-	-	HQ161800	[3]
*Singerocomus rubriflavus*	GAS 900	Brazil	KY926779	-	OM160564	-	[58]
*Singerocomus rubriflavus* (H)	TWH9585	Guyana	LC043093	-	MH645597	MH645589	[34]
*Suillellus amygdalinus*	112605ba	China	JQ326996	-	JQ327024	-	[12]
*Suillellus amygdalinus*	NY00815464	Costa Rica	KT990659	KT990484	KT990848	-	[12]
*Suillellus flaviporus*	HKAS126551	China	OQ888725	OQ873500	OQ873462	-	[12]
*Suillellus flaviporus* (H)	HKAS123826	China	OQ888726	OQ873501	OQ873463	-	[12]
*Suillellus lacrymibasidiatus* (H)	HMJAU60202	China	OM230174	OM285115	OM285117	-	[59]
*Suillellus luridus*	VDKO0241b	Belgium	-	KT824014	KT824047	KT823981	[47]
*Suillellus pinophilus* (H)	HKAS126550	China	OQ888729	OQ873504	OQ873466	-	[12]
*Suillellus queletii*	VDKO1185	Belgium	-	MH645604	MH645598	MH645590	[17]
*Suillellus subamygdalinus*	HKAS57262	China	KF112316	KF112660	KF112174	-	[12]
*Suillellus yunnanensis*	HKAS126549	China	OQ888731	OQ873506	OQ873468	-	[12]
*Suillellus yunnanensis* (H)	HKAS126548	China	OQ888730	OQ873505	OQ873467	-	[12]
*Sutorius* aff. *eximius*	HKAS52672	China	KF112399	KF112802	KF112207	-	[8]
*Sutorius* aff. *eximius*	HKAS56291	China	KF112400	KF112803	KF112208	-	[8]
*Sutorius australiensis*	REH9441	Australia	JQ327006	MG212652	JQ327032	MG212567	[46]
*Sutorius eximius*	HKAS59657	China	KT990707	KT990505	KT990887	-	[9]
*Sutorius eximius*	REH9400	USA	JQ327004	MG212653	JQ327029	MG212568	[46]
*Sutorius eximius*	HKAS50420	China	KT990549	KT990387	KT990750	-	[9]
*Sutorius* sp.	OR0378B	Thailand	-	MH614787	MH614740	MH614692	[17]
*Sutorius* sp.	OR0379	Thailand	-	MH614788	MH614741	MH614693	[17]
***Sutorius*** **sp.**	**SP18**	**China**	**PX843743**	**PX854055**	**PX880582**	**PX880586**	**This study**
*Sutorius subrufus*	FHMU2006	China	MH879699	MH879746	MH879729	-	[60]
*Sutorius subrufus* (H)	FHMU2004	China	MH879698	MH879745	MH879728	-	[60]
*Xerocomus subtomentosus*	VDKO0987	Belgium	-	MG212657	MG212614	MG212572	[46]
*Zangia citrina* (H)	HKAS52684	China	HQ326941	-	HQ326872	HQ326850	[39]
*Zangia olivacea* (H)	HKAS45445	China	HQ326945	-	HQ326873	HQ326854	[39]

### 2.3. Phylogenetic Analyses

Firstly, sequence processing was performed using Sequencher v4.1.4 (Gene Code Corp., Ann Arbor, MI, USA) to assemble sequences, remove regions of ambiguous bases, and merge degenerate bases. Then, sequences were aligned with MAFFT v7.526 using the E-INS-i strategy [61] and manually verified in BioEdit v7.7.1 [62]. Each single-gene dataset was checked for topological incongruence; Maximum Likelihood (ML) analyses were conducted on every single-gene dataset. All single-gene alignments were then concatenated using Phyutility v2.2 [63], with any missing sequence data coded as gaps, to generate a final super-matrix of all four gene loci.

The best-fit nucleotide substitution model for each partition was selected under the Akaike Information Criterion (AIC) using MrModeltest v2.3 [64]. Specifically, the GTR+I+G was the best model for LSU, *tef1-α* and *atp6*, whereas SYM+I+G was selected as the best model for *rpb2*. Phylogenetic inferences were conducted using both ML and Bayesian Inference (BI) approaches. The ML analysis was performed in RAxML v7.2.6 [65] on the combined dataset under the GTRGAMMAI model, and nodal support was assessed with 1000 non-parametric bootstrap replicates. For the BI analysis, executed in MrBayes v3.2.6 [66], we implemented a partitioned mixed model, treating the LSU, *rpb2*, *tef1-α*, and *atp6* sequences as independent partitions, each with its own model parameters. The Markov Chain Monte Carlo (MCMC) analysis was run for four million generations, sampling trees every 100 generations, with a stop rule value (STOPVAL) set at 0.01. Chain convergence was confirmed using Tracer v1.5 (http://tree.bio.ed.ac.uk/software/tracer/, accessed on 15 October 2025), ensuring all effective sample size (ESS) values exceeded 200. A 25% burn-in was applied before summarizing the posterior distribution of trees using the sump and sumt commands.

In light of the genetic relationships within Boletaceae, two closely related genera (*Buchwaldoboletus* Pilát and *Chalciporus* Bataille) were selected as outgroups. Specifically, the taxa were *Buchwaldoboletus xylophilus* (Petch) Both & B. Ortiz, *Buchwaldoboletus lignicola* (Kallenb.) Pilát, *Chalciporus piperatus* (Bull.) Bataille, and *Chalciporus africanus* Degreef & De Kesel.

## 3. Results

### 3.1. Phylogenetic Results

**Figure 1:**
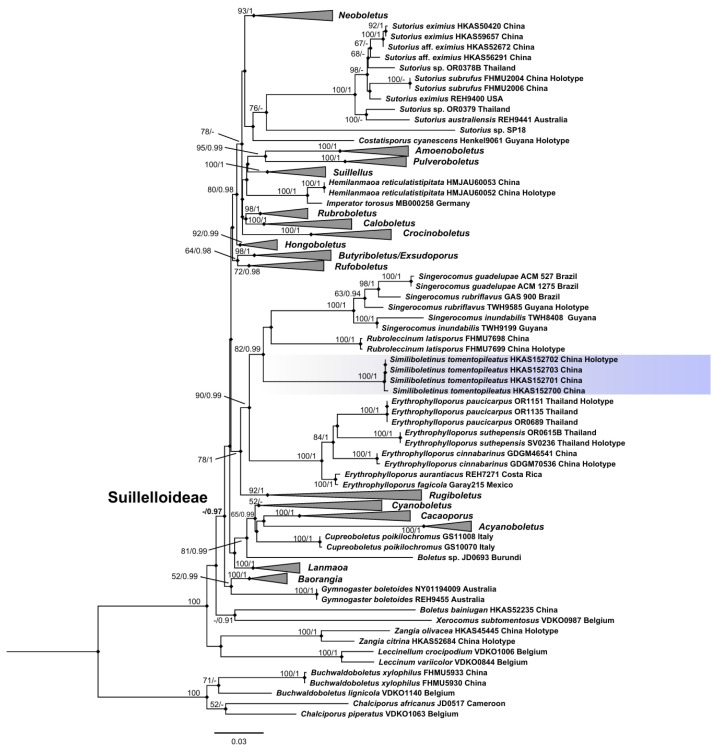
Phylogenetic relationships among representative genera in Boletaceae were inferred from a multigene dataset (LSU-*rpb2*-*tef1-α*-*atp6*) using both ML and BI methods (only shown the ML tree). Supported branches indicate bootstrap supports (BS > 50%) and posterior probabilities (PP > 0.90). Sequences from type specimens (holotype) are marked, while the new genus and new species are highlighted with a bluish background.

For the single-gene or multi-gene dataset, no topological inconsistency was detected between the ML and BI analyses. Thus, the phylogenetic tree inferred from the ML strategy is presented, with statistical results from both ML (Bootstrap Supports, BS) and BI (Posterior Probabilities, PP) displayed on the branches (see Figure 1). In the multi-gene matrix, we assembled a total of 182 collections: 133 for LSU, 170 for *rpb2*, 174 for *tef1-α*, and 56 for *atp6*. The final combined dataset included 3755 sites (including gaps): 1004 bp for LSU, 800 bp for *rpb2*, 1207 bp for *tef1-α*, and 744 bp for *atp6*.

Phylogenetic analysis placed the monophyletic new genus within Suillelloideae (BS/PP = -/0.97) with strong statistical support (BS/PP = 100/1), confirming it as both a novel genus and a novel species. It was phylogenetically closer to three genera, *viz.*, *Rubroleccinum*, *Singerocomus*, and *Erythrophylloporus*.

### 3.2. Morphological Observations and SEM

**Figure 2:**
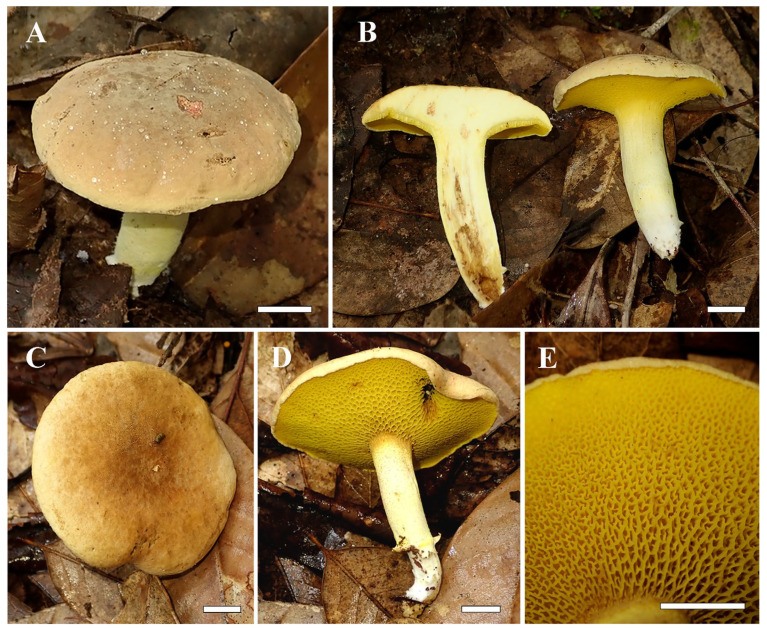
Fresh basidiomata of *Similiboletinus tomentopileatus*. KUN-HKAS 152701: (**A**) basidiomata; (**B**) context; KUN-HKAS 152702 (holotype): (**C**) pileus; (**D**) hymenophore; (**E**) pores. Bars = 1 cm.

**Figure 3:**
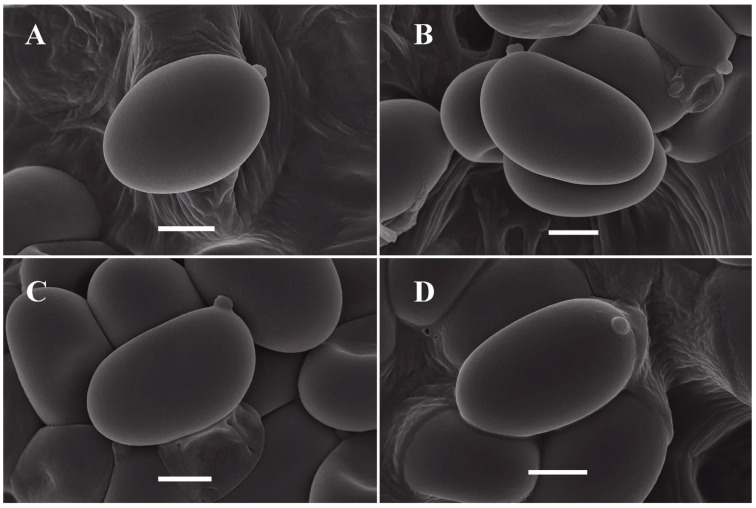
(**A**–**D**) Different views of basidiospores of *Similiboletinus tomentopileatus* (KUN-HKAS 152703) captured by SEM. Bars = 2 μm.

The images of fresh basidiomata, substrate and habitats of the collected specimens are shown in Figure 2. The scanning electron microscopy revealed that the basidiospore surface is smooth (Figure 3).

### 3.3. Taxonomy

*Similiboletinus* S.P. Jian & Yan C. Li, gen. nov.

MycoBank MB861840

Type species: *Similiboletinus tomentopileatus* S.P. Jian & Yan C. Li

Diagnosis: *Similiboletinus* differs from other genera within Suillelloideae by the following characteristics: stipitate-pileate basidiomata, a tomentose and yellow–brown pileus, a slightly thick context, a poroid hymenophore with short tubes, a white stipe base, and unchanging in color of the context, hymenophore and stipe when injured. Additionally, it also features a boletoid hymenophoral trama, a cutis-type pileipellis, and broadly ellipsoid to ovoid, smooth basidiospores (Table 3).

Etymology: “Simili-” from latin similis, similar to, because its morphological similarity to *Boletinus* Kalchbr.

Description: Basidiomata stipitate-pileate with a poroid hymenophore. Pileus plano-convex, tomentose to subtomentose; context yellowish, unchanging in color when injured. Hymenophore yellow, subdecurrent to adnate, unchanging in color when injured, slightly radially arranged. Pores compound and angular. Stipe central, yellowish to cream, interspersed with granular or punctate squamules; context cream to yellowish, unchanging in color when injured; mycelium at the base of stipe white to whitish. Basidiospores broadly ellipsoid, ellipsoid to ovoid, smooth, thin-walled, and inamyloid. Pleurocystidia and cheilocystidia present, lanceolate, lageniform, or ventricose-mucronate. Pileipellis a cutis. Clamp connections absent.

**Figure 4:**
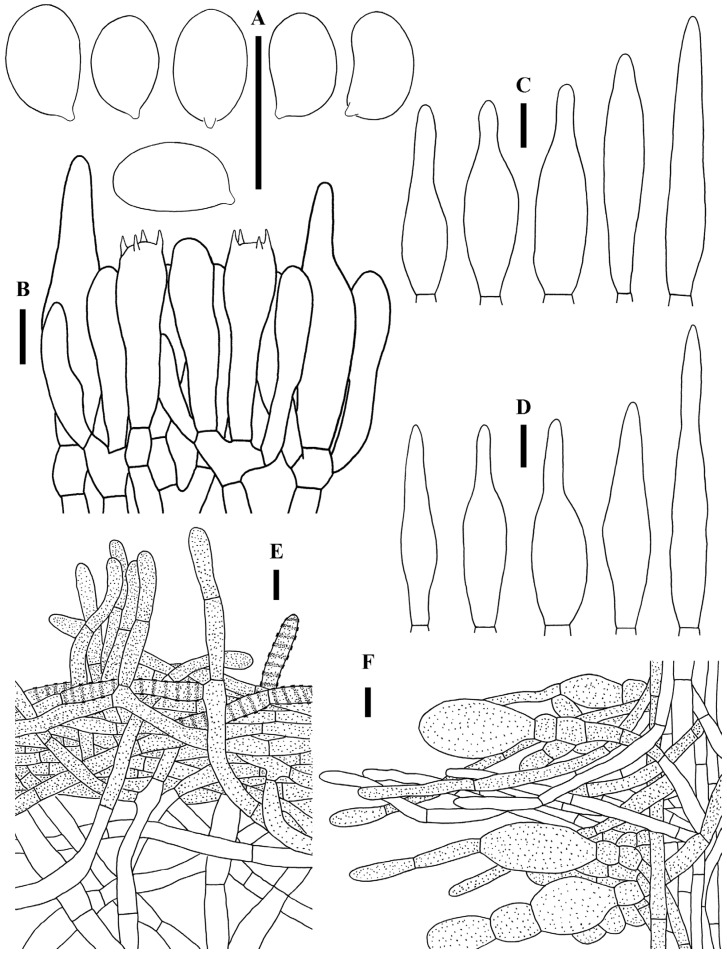
Microscopic features of *Similiboletinus tomentopileatus* (KUN-HKAS 152702, holotype). (**A**) basidiospores; (**B**) hymenium and subhymenium; (**C**) cheilocystidia; (**D**) pleurocystidia; (**E**) pileipellis; (**F**) stipitipellis. Bars: 10 μm.

*Similiboletinus tomentopileatus* S.P. Jian & Yan C. Li sp. nov. Figure 2, Figure 3 and Figure 4.

MycoBank MB861841

Holotype: China, Yunnan Province, Dai Autonomous Prefecture of Xishuangbanna, Mengla County, Yao District, Yao Ethnic Town, E 101.50, N 21.77, alt. 945 m, scattered on soil, in the tropical broadleaved forest (*Lithocarpus* and *Castanopsis*), 6 August 2025, collected by S.P. Jian, X.J. Xu, and F. Gao, JSP2025-240 (KUN-HKAS 152702). GenBank: ITS = PX843738; LSU = PX843741; *rpb2* = PX854053; *tef1-α* = PX880580; *atp6* = PX880584.

Etymology: “*tomentopileatus*” refers to the tomentose pileal surface.

Diagnosis: This new species is characterized by its yellow–brown and tomentose to subtomentose pileus, yellow subdecurrent hymenophore with compound angular pores and short tubes, and yellowish to cream stipe. It also features broadly ellipsoid to ovoid basidiospores, a boletoid hymenophoral trama, a cutis-type pileipellis with some erect hyphae, and unchanging in color in all parts when injured.

Description: Basidiomata small to medium-sized. Pileus 4.8–8.5 cm in diam., plano-convex to convex or hemispherical to subhemispherical; surface tomentose to subtomentose, hydrophobic, yellow–brown (#845432) to brown (#724311) near the center, yellowish (#efd598) to pale orange (#e5cd8b) towards the margin, sometimes uniformly yellowish (#dfc485) or brownish (#cda56d); margin incurved; context 0.7–1.2 cm thick at the center of the pileus, yellowish (#e8eeba), unchanging in color when injured. Hymenophore poroid, subdecurrent to adnate to the stipe, slightly radially arranged; tubes short, 0.2–2.0 cm in length, yellow (#d2c459), unchanging in color when injured; pores compound, angular, faveolate, yellow (#fde66f) to brilliant yellow (#c7a005), single pore around 0.5–0.8 mm, compound pore around 1–2 mm. Stipe 4.0–6.0 × 0.6–1.3 cm, central, solid but sometimes fragile, tapering downwards; surface yellowish (#e2e1a7), interspersed with dark yellow (#8c6d01) granular or punctate squamulose; context cream (#ecf1be) in the inner part, yellowish (#e7ecb5) towards the peripheral layers, unchanging in color when injured; basal mycelium white (#cbccb6) to whitish (#cacebb). Odor and taste indistinct or mild.

Basidiospores [88/4/4] 6.0–9.0 × 4.0–5.0 (5.5) μm, L_m_ × W_m_ = 7.20 (±0.60) × 4.50 (±0.23) μm, Q = 1.30–1.91 (Q_m_ = 1.55 ± 0.11), yellowish in KOH, broadly ellipsoid, ellipsoid to ovoid in side and face view, occasionally phaseoliform or allantoid in side view, smooth, thin-walled, inamyloid. Basidia 28–38 × 8–12 µm, clavate, four-spored, rarely two-spored. Cheilocystidia 40–74 × 6–14 μm, abundant, thin-walled, lanceolate, lageniform or ventricose-mucronate. Pleurocystidia 47–75 × 9–14 μm, abundant, thin-walled, lanceolate, lageniform or ventricose-mucronate. Hymenophoral trama boletoid, consisting of a compact central strand of cylindrical to subcylindrical hyphae (4–22 μm wide) with sparse, gelatinous hyphae diverging from center towards the subhymenium. Pileipellis a cutis, around 45–104 μm thick, composed of filamentous, cylindrical hyphae, 4.5–10.5 μm wide, smooth or incrusted, containing yellowish to yellow pigments either intracellular or parietal. Pileal trama composed of interwoven or irregularly arranged, hyaline, cylindrical hyphae, 4.5–16.5 μm wide. Stipitipellis a cutis composed of yellowish hyphae, 3–13 μm wide; sometimes with hyaline or yellowish, filamentous erect hyphae, 5–13 μm wide; occasionally intermixed with yellowish, inflated, globular to clavate terminal cells measuring 16–43 × 11–26 μm. Stipe trama composed of regular, compact, thin-walled, parallel hyphae, 4–13 μm wide. Clamp connections absent.

Ecology and distribution: Solitary, scattered on soil under tropical fagaceous trees (mainly *Lithocarpus* and *Castanopisis*), distributed in Yunnan Province, China, June to August.

Additional specimens examined: China, Yunnan Province, Dai Autonomous Prefecture of Xishuangbanna, Mengla County, Yao District, Yao Ethnic Town, E 101.50, N 21.77, alt. 945 m, scattered on soil, in the tropical broadleaved forest (*Lithocarpus* and *Castanopsis*), 30 June 2025, collected by S.P. Jian, X.J. Xu, and Y.W. Fang, JSP2025-107 (KUN-HKAS 152700); *ibid.*, alt. 945 m, scattered on soil, in the tropical broadleaved forest (*Lithocarpus* and *Castanopsis*), 6 August 2025, collected by S.P. Jian, X.J. Xu, and F. Gao, JSP2025-239 (KUN-HKAS 152701); *ibid.*, alt. 943 m, scattered on soil, in the tropical broadleaf (*Lithocarpus* and *Castanopsis*) forest, 6 August 2025, collected by S.P. Jian, X.J. Xu, and F. Gao, JSP2025-241 (KUN-HKAS 152703).

Notes: In the phylogenetic tree, *S. tomentopileatus* belongs to Suillelloideae. However, this new taxon is morphologically more similar to *Boletinus asiaticus* Singer, *Boletinellus merulioides* (Schwein.) Murrill, *Chalciporus radiatus* Ming Zhang & T.H. Li, *Hemileccinum parvum* Mei-Xiang Li et al., *Singerocomus inundabilis*, *Suillus americanus* (Peck) Snell, and *Xerocomus yunnanensis* (W.F. Chiu) F.L. Tai. *Boletinus asiaticus*, first described from Siberia, form obligate ECM association with *Larix* spp. in boreal regions and is characterized by radially arranged and yellow hymenophore. It differs from *S. tomentopileatus* by its noteworthy purple–red pileus, the presence of a veil, annulus, and hollow stipe [67,68]. Another species similar to *S. tomentopileatus* is *Boletinellus merulioides*, which has a yellow–brown pileus and a deeply decurrent hymenophore and was originally reported from North America. It could be distinguished from *S. tomentopileatus* by its short and eccentric stipe, ovoid or subglobose basidiospores (7–10 × 6–7.5 μm) and gregarious growth on stumps and decaying roots [10,30]. *Chalciporus radiatus*, originally described from Hunan Province, China, is characterized by its brownish orange pileus and a radially arranged hymenophore with short tubes. However, it differs from *S. tomentopileatus* in its yellowish white context, turning pastel yellow to light yellow when injured, brown to reddish-brown hymenophore, and fusiform to cylindrical basidiospores (Q_m_ = 2.12) [69].

*Hemileccinum albidum* has a brownish pileus and no color change in context when injured. However, it differs from *S. tomentopileatus* by its hymenophore depressed around the stipe, much longer stipe (5–16 cm), and larger basidiospores (11–12.5 × 4.5–5.5 μm) [70]. *Singerocomus inundabilis* was initially discovered in Brazil and placed in the genus *Xerocomus* Quél. as *Xerocomus inundabilis* Singer. Then, Henkel et al. [34] proposed a new genus, *Singerocomus*, to accommodate this species. It is characterized by a pinkish-red pileus and stipe, as well as an unchanging color of the hymenophore when injured. This species differs from *S. tomentopileatus* in its hymenophore depressed around the stipe, phylloporoid hymenophoral trama, and olivaceous-brown basidiospores. *Suillus americanus* could be confused with *S. tomentopileatus* due to its yellow pileus and adnate hymenophore. However, it can be distinguished by its viscid pileal surface, slow color change in hymenophore when injured, and relatively large basidiospores (8–11 × 3–4 μm) [10,71]. Lastly, *Xerocomus yunnanensis*, originally described from Yunnan Province, China, also resembles *S. tomentopileatus* in its yellowish brown and tomentose pileus and slightly decurrent hymenophore. Nonetheless, it differs significantly from *S. tomentopileatus* due to its very small-sized basidiomata (only 1.5–2 cm), larger basidiospores (9–12 × 4–5 μm), and trichoderm pileipellis [72,73].

## 4. Discussion

Bolete fungi of Yunnan Province, China—a globally recognized biodiversity hotspot [74]—are well known for their edible and medicinal value [9,75,76,77]. The determination of the new genus *Similiboletinus* in this region further underscores the remarkable diversity of the Boletaceae in Yunnan. In this study, phylogenetic analyses reveal that *Similiboletinus* is closely related to *Erythrophylloporus*, *Rubroleccinum*, *Rugiboletus*, and *Singerocomus*. *Erythrophylloporus*, established by Zhang and Li [23], is characterized by a yellowish-orange to red pileus and a lamellate hymenophore. Such traits clearly differentiate it from *Similiboletinus*. The recently described genus *Rubroleccinum* is characterized by a red-tinged pileus, a color-changing hymenophore upon injury, and a trichoderm pileipellis [32]. In contrast, *Rugiboletus* typically exhibits larger basidiomata (pileus size > 9 cm), long basidiospores (length > 10 μm), and trichoderm pileipellis (such as trichoderm, ixotrichoderm, and so on) [15,55,78]. Species of *Singerocomus* are mainly distributed in neotropical regions such as Brazil and Guyana [34,57,79] and are characterized by pinkish-red to red pileus and stipe, hymenophore depressed around the stipe, phylloporoid hymenophoral trama, and trichoderm pileipellis [34]. Despite the phylogenetic proximity, the aforementioned genera are clearly distinct from *Similiboletinus* in key morphological characteristics, contrasting with other genera (below) that exhibit greater morphological convergence.

Morphologically, the basidiomata of *Similiboletinus* may be confused with those of *Acyanoboletus*, *Boletinus*, *Boletinellus* Murrill, *Hemileccinum*, and *Xerocomus*. *Acyanoboletus*, recently proposed by Wu et al. [12], is distinguished by its strongly incurved pileal margin, strong unpleasant odor, and intricate trichoderm pileipellis, features absent in *Similiboletinus*. *Boletinus* was erected by Kalchbrenner [80] and characterized by Singer [5], who noted the presence of clamp connections. In morphology, *Boletinus* is strictly associated with conifer (especially larch trees) and shares with *Similiboletinus* a yellow hymenophore decurrent around the stipe. However, it differs from *Similiboletinus* by its membranous annulus and clamp connections [5]. *Boletinellus* could be confused with *Similiboletinus* due to its tomentose pileus and radially arranged hymenophore decurrent around the stipe. However, it could significantly differ from *Similiboletinus* by its brown pileus with fulvous or olivaceous tinge and clamp connections [10,81]. *Hemileccinum* was established by Šutara [82] based on morphological analysis and later confirmed phylogenetically by Nuhn et al. [83] and Wu et al. [8]. While *Hemileccinum* shares with *Similiboletinus* the absence of color change upon injury and yellow hymenophore, it differs in its adnate to sinuate hymenophore, long (length > 10 μm), cylindrical (Q_m_ > 2), and irregularly warty basidiospores (observed under SEM), and trichoderm peileipellis. Finally, *Xerocomus* is a historically recognized genus [82], and its circumscription (i.e., *Xerocomus* sensu stricto) has been refined through phylogenetic studies [9,72,83]. Its adnate or sinuate hymenophore, color-changing context when injured, mostly bacillate basidiospores, and trichoderm peileipellis make it different from *Similiboletinus*.

## 5. Conclusions

In summary, the newly proposed genus *Similiboletinus* is clearly distinguished from other known genera in Boletaceae by a unique combination of morphological characters: a yellow–brown pileus, a subdecurrent yellow hymenophore with compound angular pores, a yellowish to cream stipe, broadly ellipsoid to ovoid basidiospores, a boletoid hymenophoral trama, a cutis-type pileipellis with some erect hyphae, and no color change in any part of the basidiomata when injured (see detail in Table 3). Although only one species of *Similiboletinus* has been identified in this study, more extensive fieldwork and integrative taxonomic research are likely to reveal more species diversity within this new genus. Furthermore, while nearly 400 bolete taxa have been recorded in China to date, it is anticipated that many more remain to be discovered in the future.

## Figures and Tables

**Table 1:** Genera of the subfamily Suillelloideae supported by morphological and molecular phylogenetic analyses (as of December 2025).

Genera	Type Species	Type Locality	References
*Acyanoboletus* G. Wu & Zhu L. Yang *	*Acyanoboletus controversus* G. Wu & Zhu L. Yang	Yunnan, China	[12]
*Amoenoboletus* G. Wu et al. *	*Amoenoboletus granulopunctatus* (Hongo) G. Wu et al.	Honshu, Japan	[14]
*Baorangia* G. Wu & Zhu L. Yang *	*Baorangia pseudocalopus* (Hongo) G. Wu & Zhu L. Yang	Kyoto, Japan	[15]
*Butyriboletus* D. Arora & J.L. Frank	*Butyriboletus appendiculatus* (Schaeff.) D. Arora & J.L. Frank	Bavaria, Germany	[16]
*Cacaoporus* Raspé & Vadthanarat	*Cacaoporus tenebrosus* Vadthanarat et al.	Chiang Mai, Thailand	[17]
*Caloboletus* Vizzini	*Caloboletus calopus* (Pers.) Vizzini	Bavaria, Germany	[18]
*Costatisporus* T.W. Henkel & M.E. Sm.	*Costatisporus cyanescens* T.W. Henkel & M.E. Sm.	Guyana	[19]
*Crocinoboletus* N.K. Zeng et al. *	*Crocinoboletus rufoaureus* (Massee) N.K. Zeng et al.	Singapore	[20]
*Cupreoboletus* Simonini et al.	*Cupreoboletus poikilochromus* (Pöder et al.) Simonini et al.	Emilia-Romagna, Italy	[21]
*Cyanoboletus* Gelardi et al.	*Cyanoboletus pulverulentus* (Opat.) Gelardi et al.	Berlin, Germany	[22]
*Erythrophylloporus* Ming Zhang & T.H. Li *	*Erythrophylloporus cinnabarinus* Ming Zhang & T.H. Li	Hainan, China	[23]
*Exsudoporus* Vizzini et al.	*Exsudoporus permagnificus* (Pöder) Vizzini et al.	Sardegna, Italy	[24]
*Gymnogaster* J.W. Cribb	*Gymnogaster boletoides* J.W. Cribb	Queensland, Australia	[25]
*Hemilanmaoa* Yang Wang et al. *	*Hemilanmaoa reticulatistipitata* Yang Wang et al.	Guizhou, China	[26]
*Hongoboletus* G. Wu & Zhu L. Yang *	*Hongoboletus ventricosus* (Taneyama & Har. Takah.) G. Wu & Zhu L. Yang	Honshu, Japan	[12]
*Imperator* Koller et al.	*Imperator torosus* (Fr.) Assyov et al.	Sauvabelin, Switzerland	[27]
*Lanmaoa* G. Wu & Zhu L. Yang *	*Lanmaoa asiatica* G. Wu & Zhu L. Yang	Yunnan, China	[15]
*Neoboletus* Gelardi et al.	*Neoboletus luridiformis* (Rostk.) Gelardi et al.	Germany	[28]
*Pseudobaorangia* D.F. Sun et al.	*Pseudobaorangia lakhanpalii* (K. Das et al.) D.F. Sun et al.	Sikkim, India	[29]
*Pulveroboletus* Murrill	*Pulveroboletus ravenelii* (Berk. & M.A. Curtis) Murrill	South Carolina, USA	[30]
*Rubroboletus* Kuan Zhao & Zhu L. Yang *	*Rubroboletus sinicus* (W.F. Chiu) Kuan Zhao & Zhu L. Yang	Yunnan, China	[31]
*Rubroleccinum* N.K. Zeng et al. *	*Rubroleccinum latisporum* N.K. Zeng et al.	Fujian, China	[32]
*Rufoboletus* N.K. Zeng & Zhi Q. Liang *	*Rufoboletus hainanensis* (N.K. Zeng et al.) N.K. Zeng & Zhi Q. Liang	Hainan, China	[33]
*Rugiboletus* G. Wu & Zhu L. Yang *	*Rugiboletus extremiorientalis* (Lj.N. Vassiljeva) G. Wu & Zhu L. Yang	Vladivostok, Russia	[15]
*Singerocomus* T.W. Henkel & M.E. Sm.	*Singerocomus inundabilis* (Singer) T.W. Henkel	Amazonas, Brazil	[34]
*Suillellus* Murrill	*Suillellus luridus* (Schaeff.) Murrill	Germany	[30]
*Sutorius* Halling et al.	*Sutorius eximius* (Peck) Halling et al.	Vermont, USA	[35]

Notes: * genus described by Chinese researchers.

**Table 3:** Comparison of related genera to *Similiboletinus* in morphology and phylogeny.

Genus	Pileus	Context	Hymenophore	Hymenophoral Trama	Basidiospores	Pileipellis
*Acyanoboletus*	Brownish orange to reddish brown	Unchanging in color when injured	Poroid, sinuate to emarginate, pale yellow, unchanging in color when injured	Intermediate between phylloporoid and boletoid	Subfusiform to fusiform, smooth	Trichoderm
*Boletinus*	Red, bright pinkish, to yellowish brown	Unchanging	Poroid, adnate to decurrent, yellow, radially arranged, unchanging in color	Boletoid	Oblong to ellipsoid, smooth	Trichoderm
*Boletinellus*	Brown with olivaceous tinge	Unchanging	Poroid, decurrent, olivaceous brown, unchanging or staining blue slowly when injured	Boletoid	Shortly ellipsoid to subreniform, smooth	Trichoderm
*Erythrophylloporus*	Yellowish orange to red	Mostly slowly blackish blue to reddening when injured	Lamellate, decurrent, yellowish orange to orange, becoming grayish blue to grayish green when injured	Phylloporoid	Ellipsoid, broadly ellipsoid to ovoid, smooth	Subcutis, cutis to trichoderm
*Hemileccinum*	Yellow, brown to reddish orange	Unchanging in color when injured	Poroid, adnate to sinuate, light yellow to yellow, unchanging in color when injured	Boletoid or phylloporoid	Subfusiform to inequilateral, with tiny warts under SEM	Trichoderm, epithelium to hyphoepithelium
*Rubroleccinum*	Reddish-orange to grayish-yellow	Changing blue then red when injured	Poroid, depressed around the stipe, brilliant yellow to yellow, changing blue then red when injured	Unknown	Fusiform to cylindrical, smooth	Trichoderm
*Rugiboletus*	Yellowish, yellow to yellowish brown	Unchanging or staining blue or dark blue when injured	Poroid, adnexed, yellowish, yellow to reddish brown, unchanging or staining blue to dark blue quickly when injured	Boletoid	Subfusiform to subcylindrical, smooth	Intricate trichoderm to ixotrichoderm
*Similiboletinus*	Yellow to brown	Unchanging in color when injured	Poroid, subdecurrent to adnate, yellow, unchanging in color when injured	Boletoid	Broadly ellipsoid to ovoid	Cutis
*Singerocomus*	Pinkish red to red	Unchanging in color when injured	Poroid, depressed around the stipe, yellow, unchanging in color when injured	Phylloporoid	Ellipsoid to subfusiform, smooth	Trichoderm
*Xerocomus*	Yellowish brown to brown	Staining bluish slowly or indistinctly when injured	Poroid, adnate to sinuate, yellowish to yellow, staining blue when injured	Phylloporoid	Subfusiform	Trichoderm

## Data Availability

The sequence data generated in this study can be obtained from NCBI GenBank (http://www.ncbi.nlm.nih.gov/). The data included in this study are available by contacting the authors.
